# Supplementary material for: Interactions between lineage‐associated transcription factors govern haematopoietic progenitor states
Source: EMBO J. 2020 Oct 26;39(24):e104983. doi: 10.15252/embj.2020104983 (PMC7737608; doi:10.15252/embj.2020104983)
Supplement: Supplementary file 1 — Appendix [file EMBJ-39-e104983-s001.pdf]

Appendix

Table of contents

Appendix Figure S1 2

Appendix Figure S2 3

Appendix Figure S3 4

Appendix Figure S4 5

Appendix Figure S5 6

Appendix Figure S6 7

Appendix Figure S7 8

Appendix Figures S8-13 9

Appendix Figures (S1-S13)

Appendix Figure S1

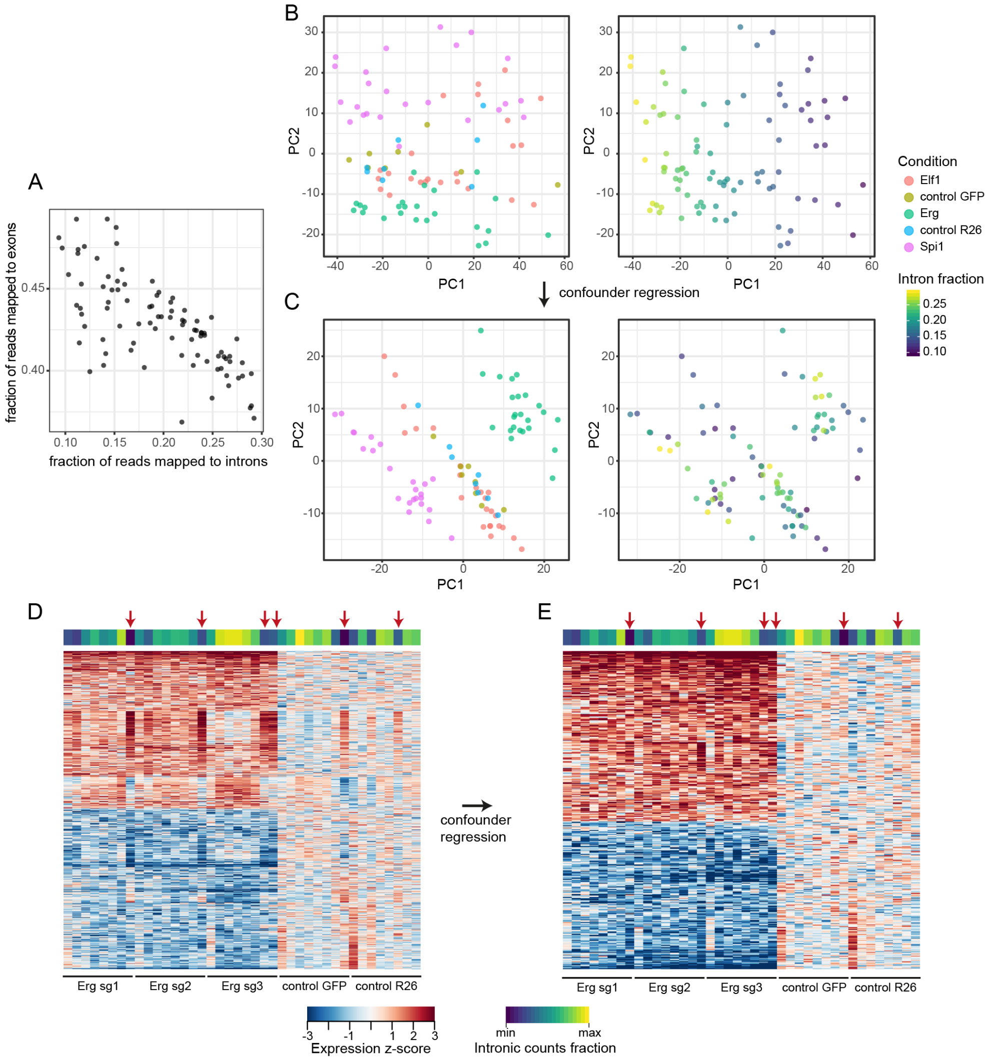

Appendix Figure S1

A Relation between the fraction of reads mapping to exons and introns, each dot represents a sample in a representative plate.

B Principal component analysis of samples within a plate, based on $\log_2$(normalised counts + 1) values. Please note that samples segregate according to the fraction of intronic reads and the experimental design.

C as in (B) but using residuals from a linear regression with intronic reads as a covariate.

D Heatmap of z-score standardised expression values for genes differentially expressed between Erg sgRNA treated cells and the controls.

E as in (D) but using residuals from a linear regression with intronic reads as a covariate. Please note that the z-score is centred around mean of the controls (i.e. expected expression value is 0). Red arrows indicate instances, where regression successfully removed unwanted variation between samples.

Appendix Figure S2

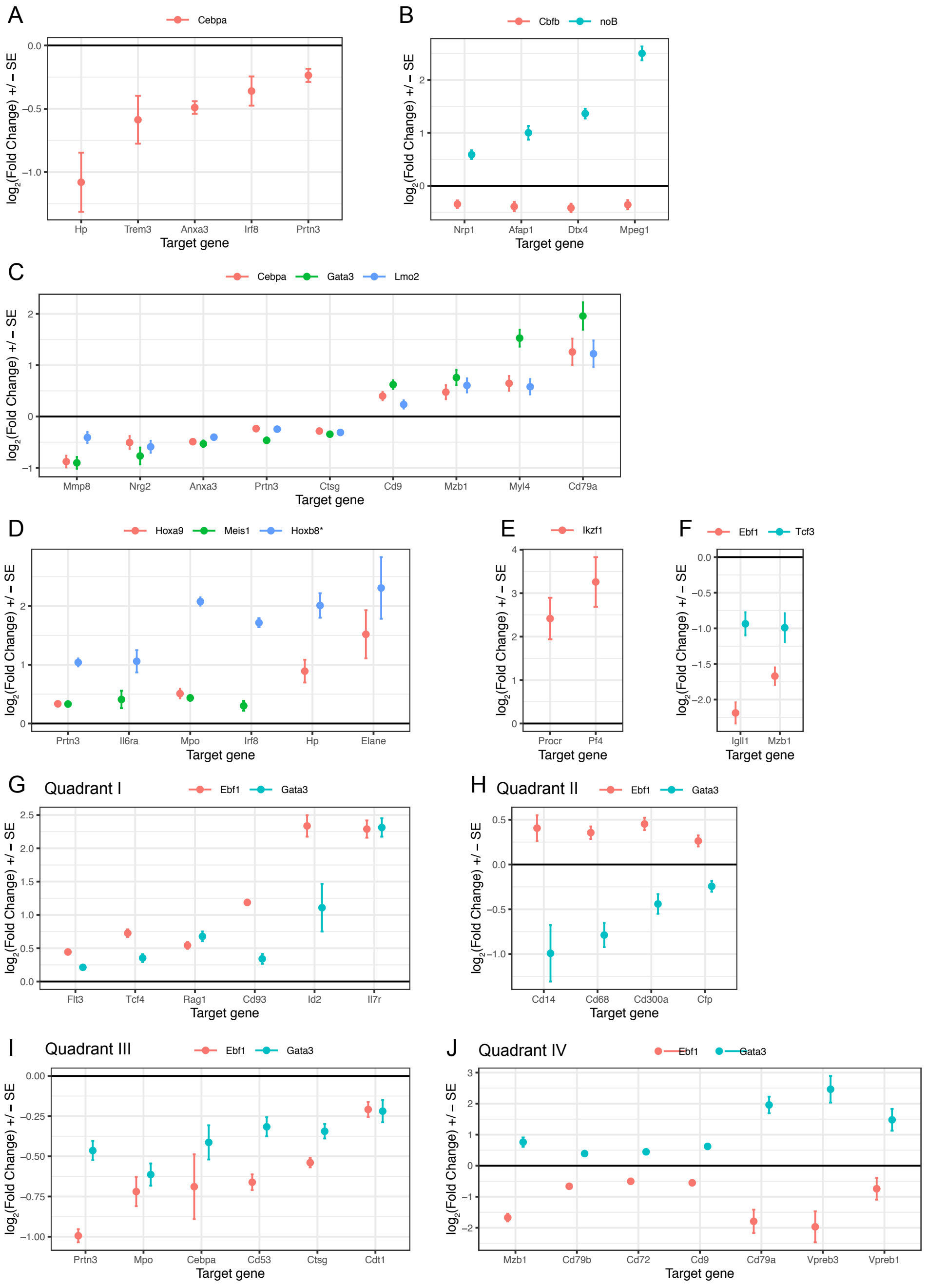

Appendix Figure S2.

A-J Observed changes in expression (mean +/- standard error of the mean) for example genes following loss of indicated TF.

Appendix Figure S3

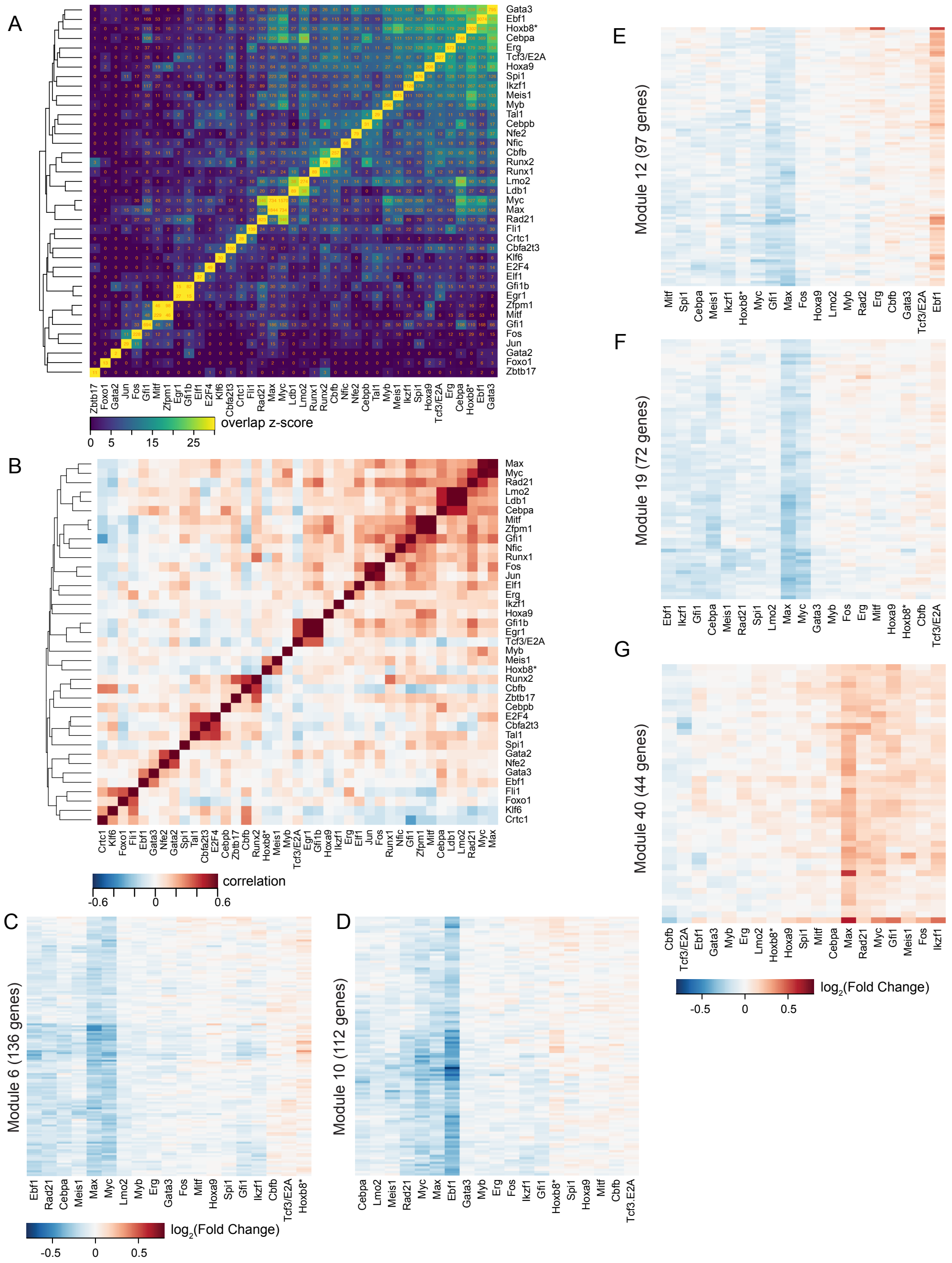

Appendix Figure S3.

A Heatmap of target genes overlap among all perturbed TFs in this study. Colour corresponds to z-scores based on the hypergeometric distribution, numbers indicate the total number of genes in each intersection. Samples were hierarchically clustered.

B Correlations in changes of gene expression following the perturbation of each TF in this study. Correlation was calculated using log_2(fold change) values adjusted for p-values for all genes expressed in Hoxb8-FL cells.

C-G Heatmaps displaying log$_2$(fold change) values adjusted for p-value for genes belonging to chosen modules (see Figure 3D). Displaying 19 TFs with >200 targets identified.

Appendix Figure S4

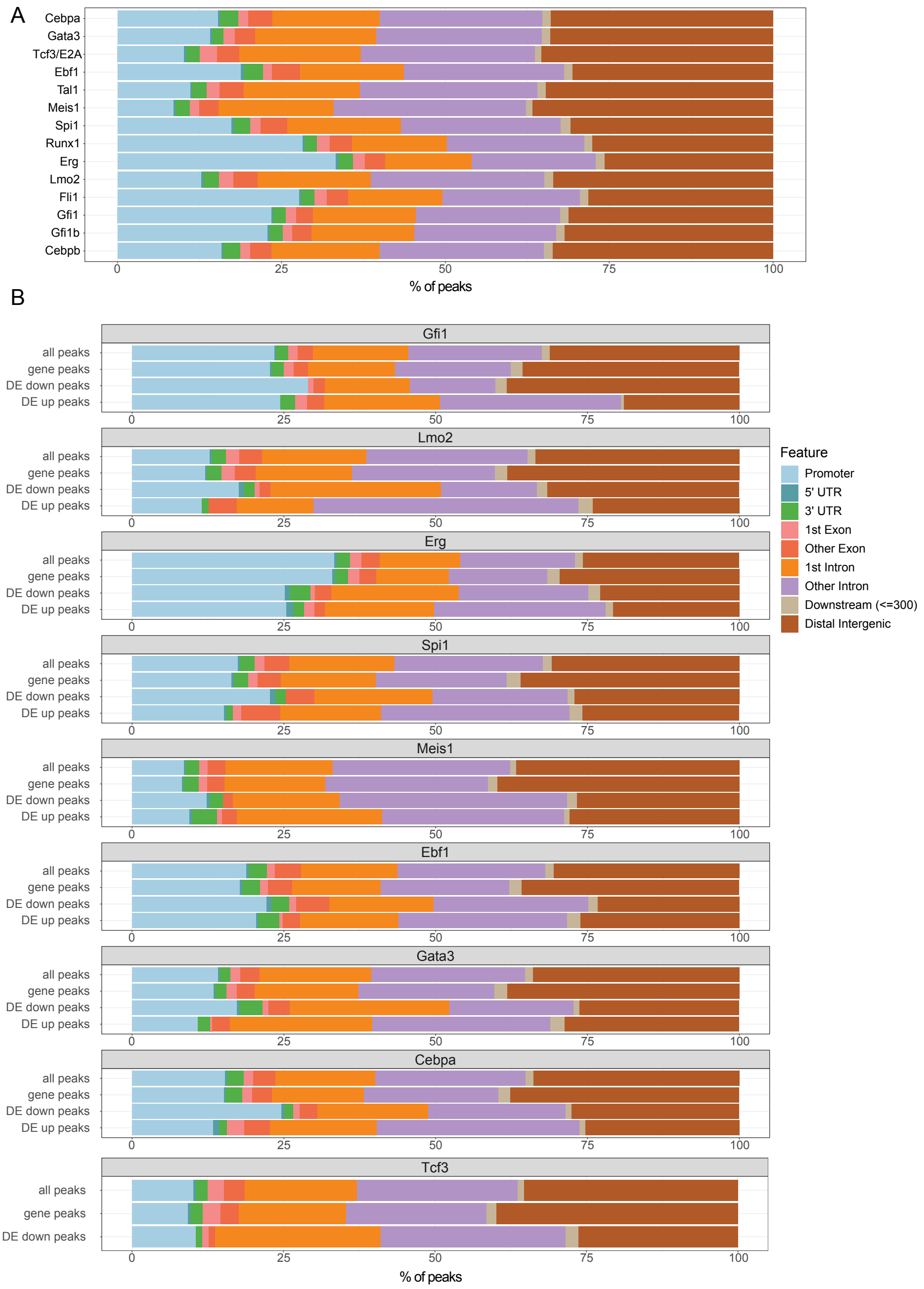

Appendix Figure S4

A Genomic distribution of transcription factor binding sites for all assayed TFs.

B Genomic distribution of transcription factor binding for four categories of peaks: all peaks identified by ChIP-Seq, peaks mapped to genes, peaks mapped to gene that are

differentially expressed (up- or down-regulated) and following loss of a corresponding TF. In case of Tcf3, the upregulated category was omitted due to a low number of observed genes.

Appendix Figure S5

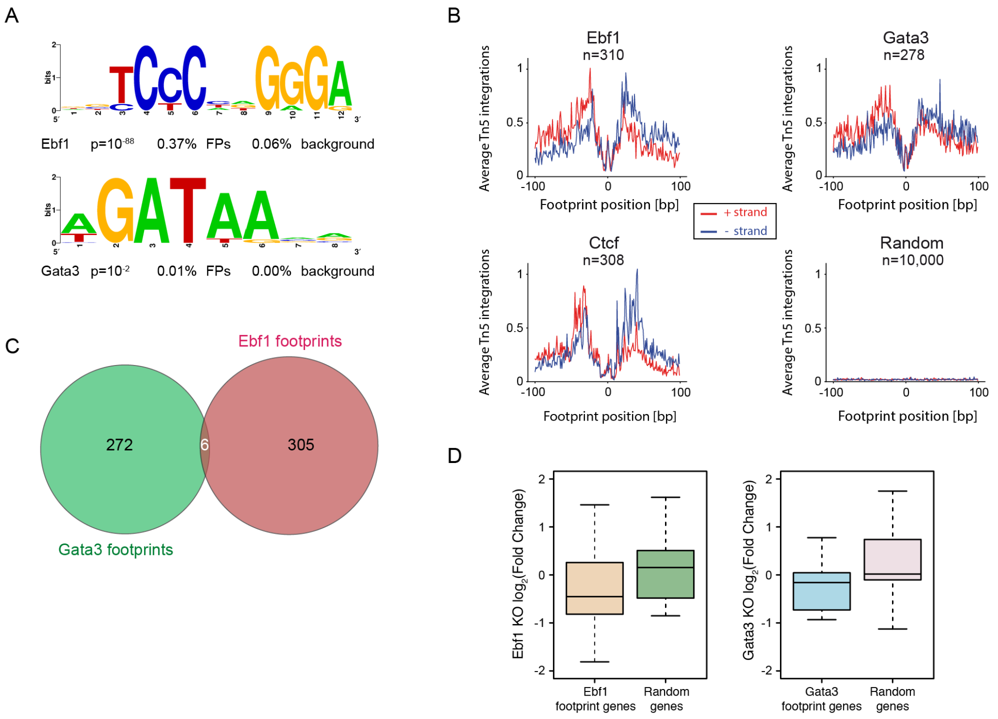

Appendix Figure S5

A Gata3 and Ebf1 motif sequence logo with enrichment statistics for Hoxb8-FL ATAC-Seq footprints.

B Relative Tn5 insertion profiles around each footprint for Ebf1, Gata3 footprints as well as Ctcf (positive control) and 10,000 random genomic regions (negative control). Positive and negative strands represented by red and blue lines,respectively.

C Overlap of genes with annotated Gata3 and Ebf1.

D Changes in expression following Ebf1 and Gata3 perturbation observed in genes with Ebf1 and Gata3 footprints respectively; compared to a random sample of genes as a control.

Appendix Figure S6

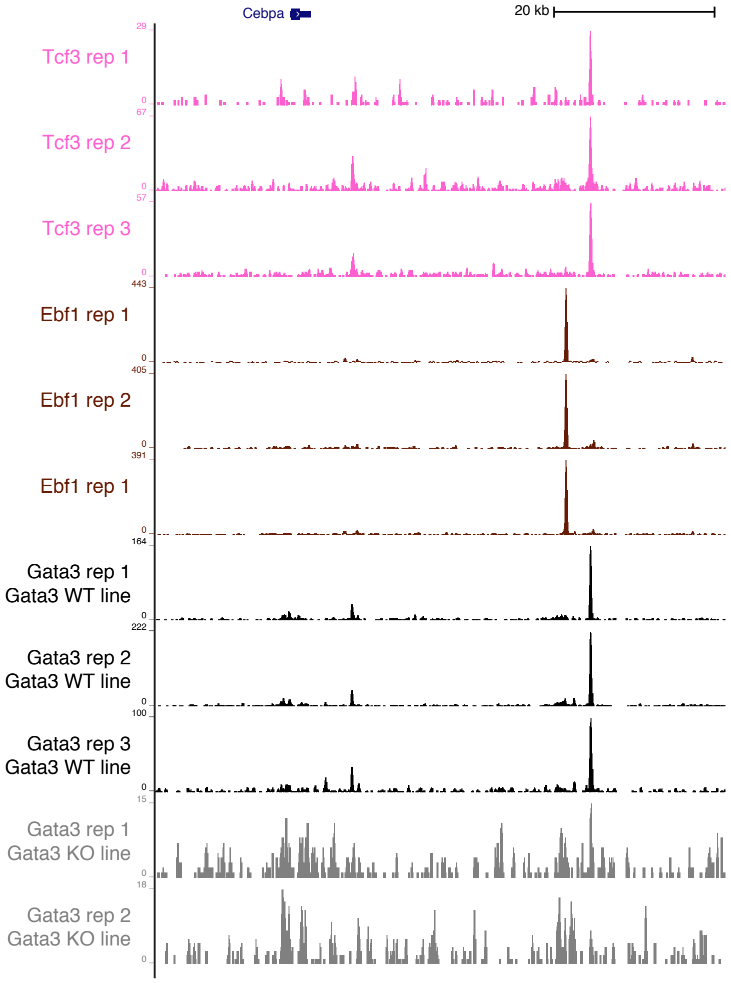

Appendix Figure S6

A genomic browser view of Cebpa locus displaying the ChIP-Seq data used in this study.

Appendix Figure S7

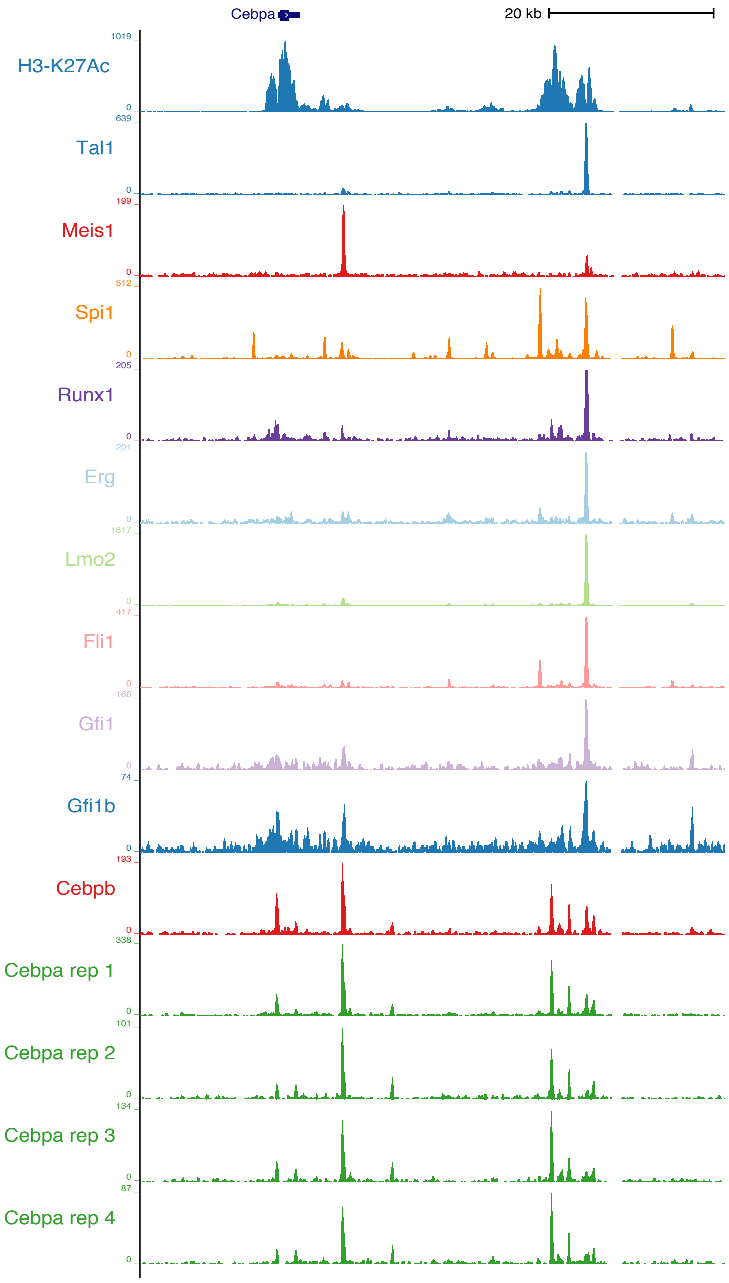

Appendix Figure S7

A genomic browser view of Cebpa locus displaying the ChIP-Seq data used in this study.

Figure S8.

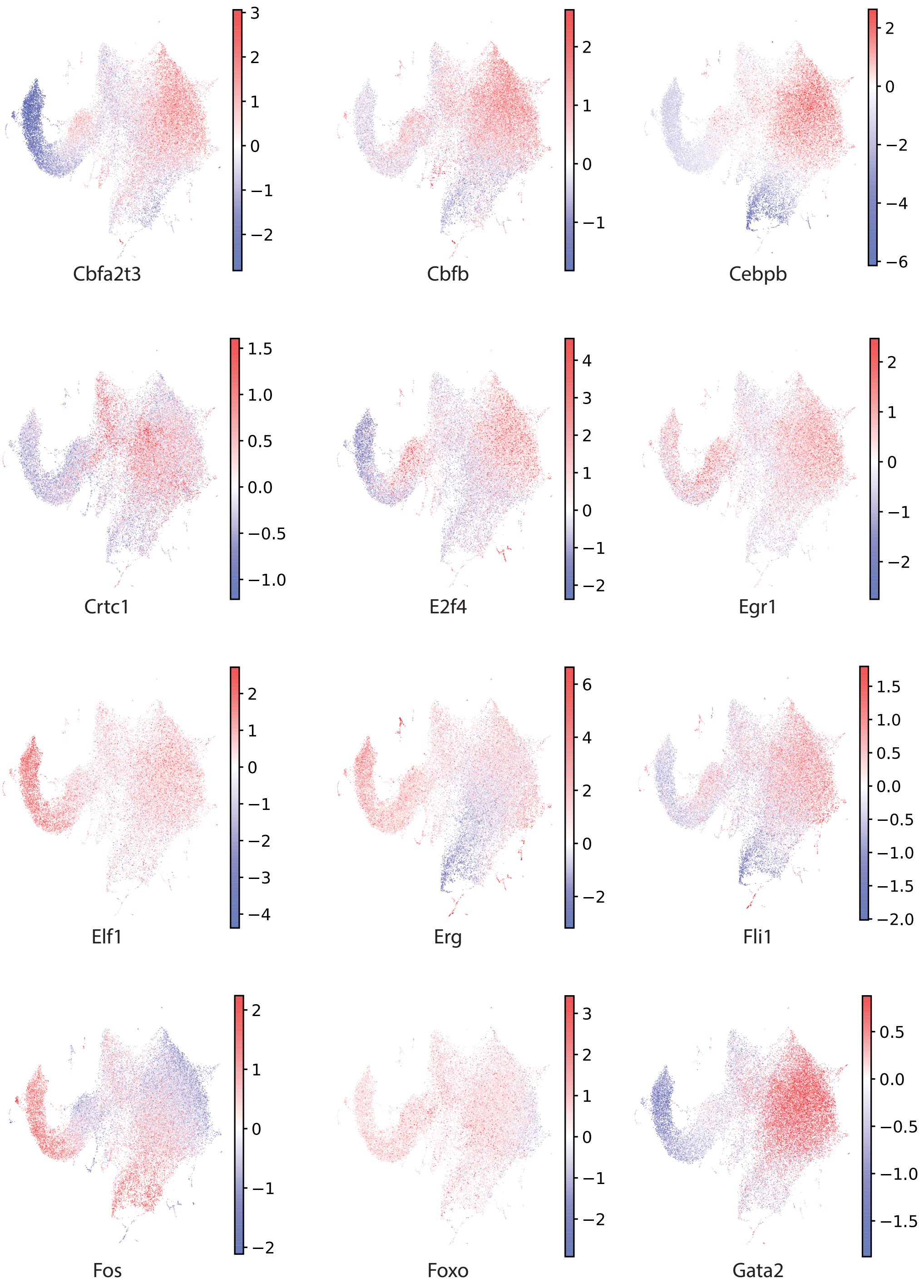
Cbfa2t3
3
2
1
0
−1
−2
Cbfb
2
1
0
−1
Cebpb
2
0
−2
−4
−6
Crtc1
1.5
1.0
0.5
0.0
−0.5
−1.0
E2f4
4
3
2
1
0
−1
−2
Egr1
2
1
0
−1
−2
Elf1
2
1
0
−1
−2
−3
−4
Erg
6
4
2
0
−2
Fli1
1.5
1.0
0.5
0.0
−0.5
−1.0
−1.5
−2.0
Fos
2
1
0
−1
−2
Foxo
3
2
1
0
−1
−2
Gata2
0.5
0.0
−0.5
−1.0
−1.5

Figure S9.

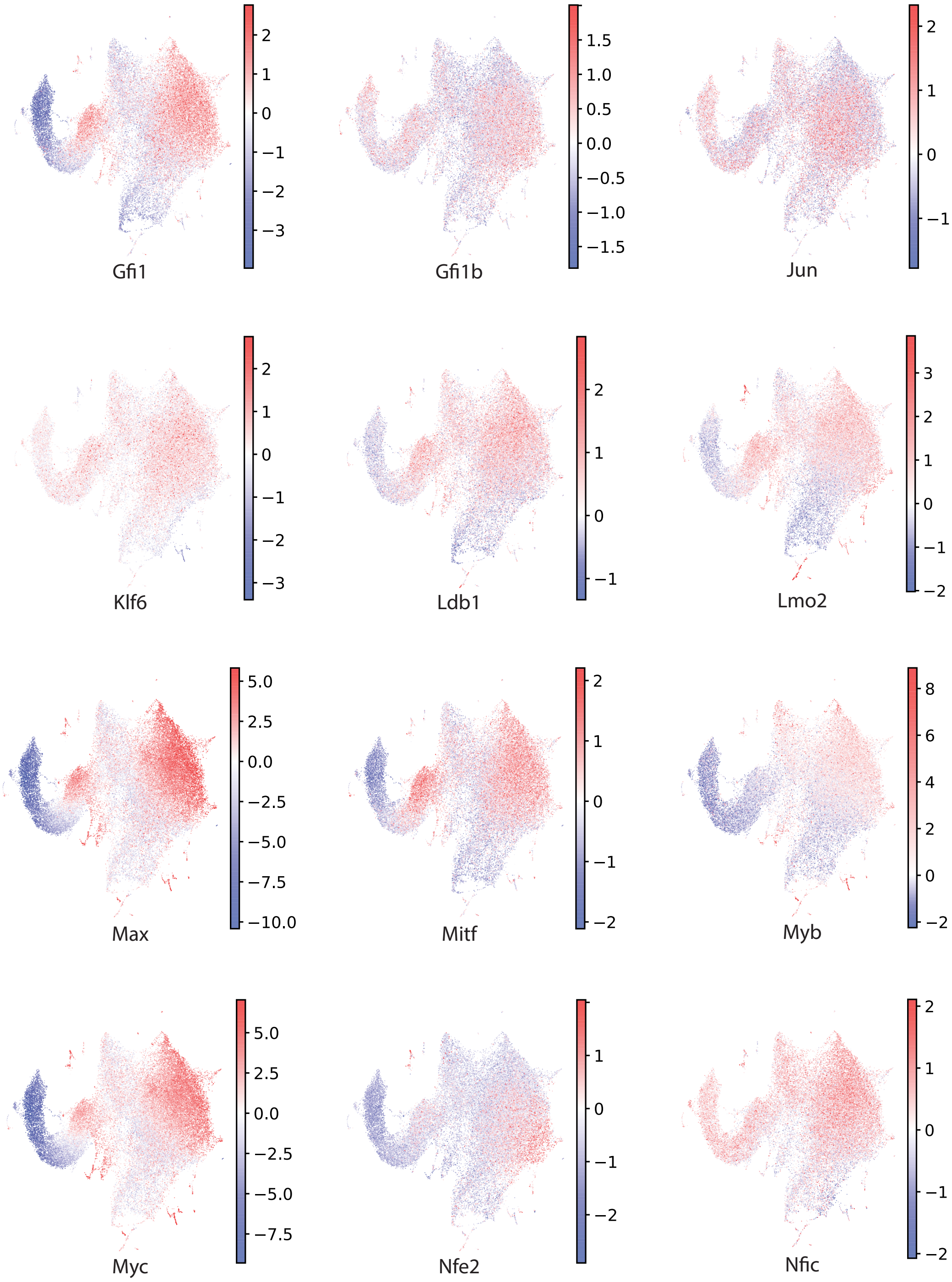

Figure S10.

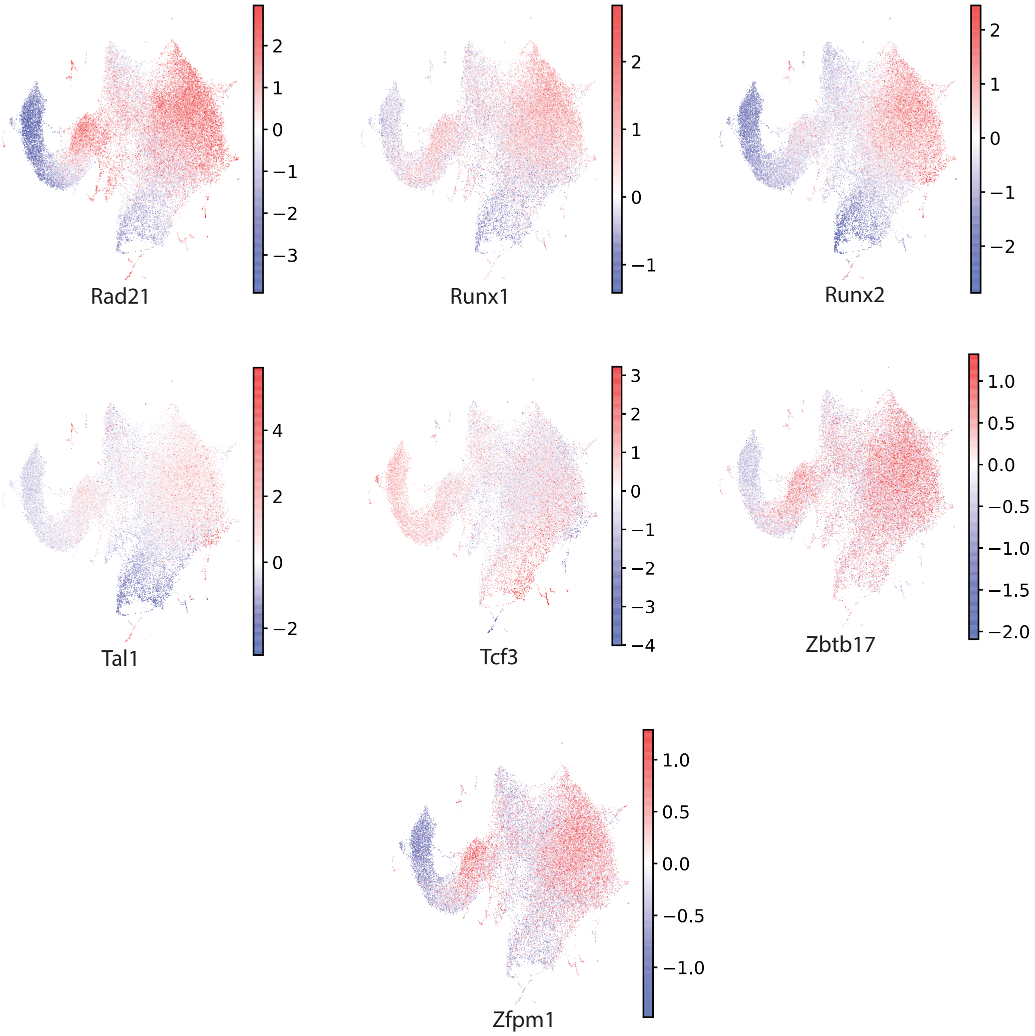

Figure S11.

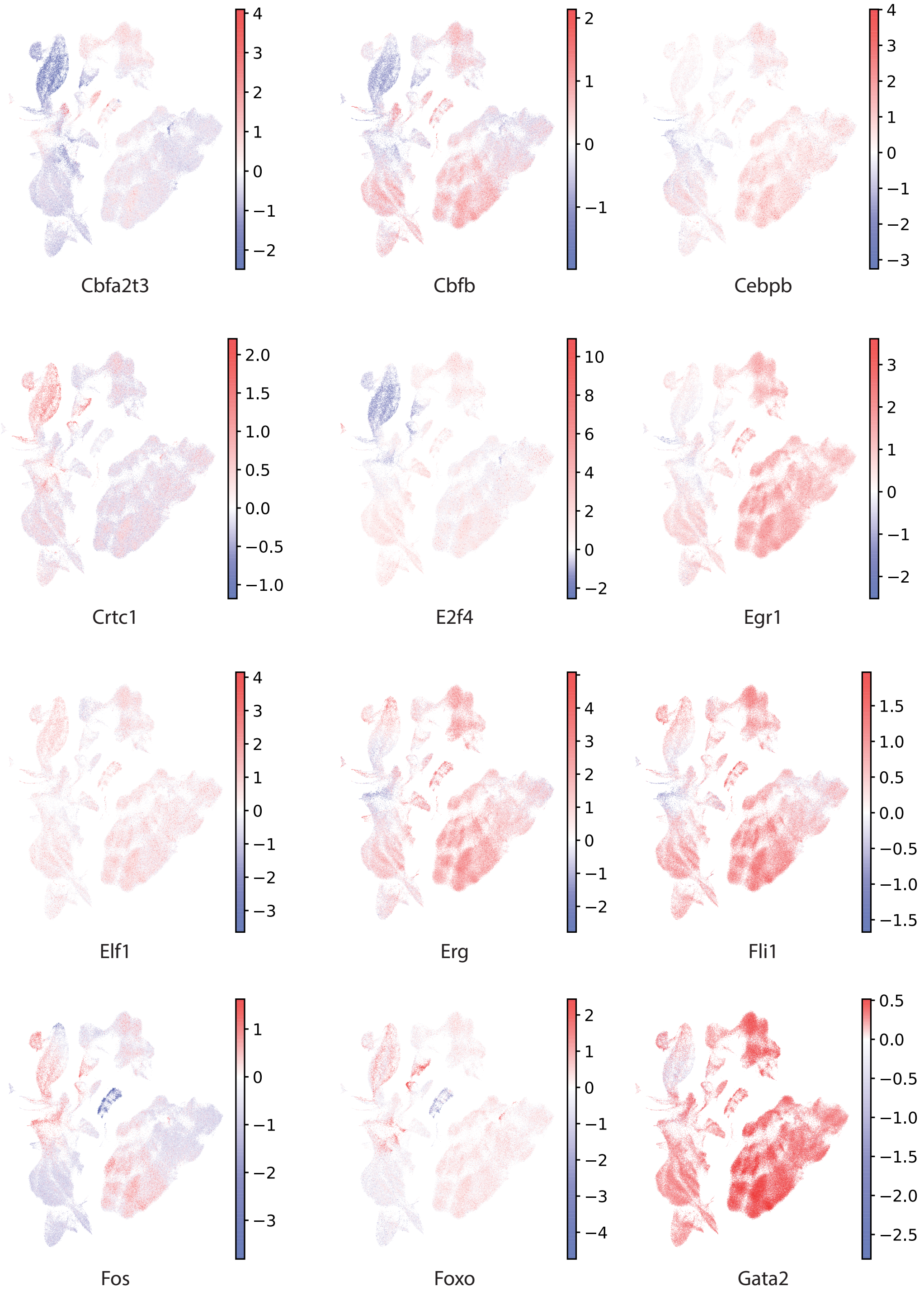

Cbfa2t3
Cbfb
Cebpb
Crtc1
E2f4
Egr1
Elf1
Erg
Fli1
Fos
Foxo
Gata2

Figure S12.

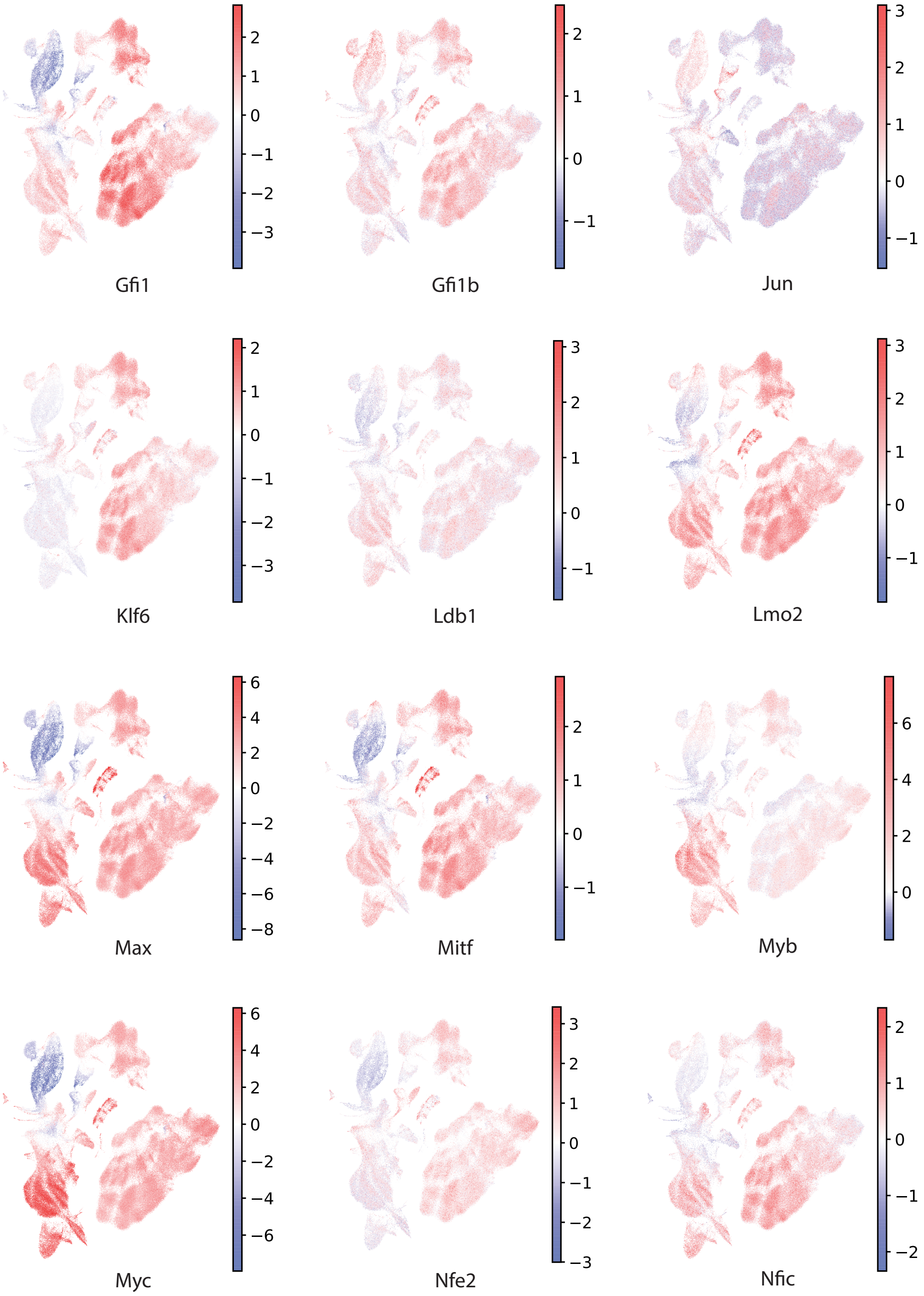

Figure S13.

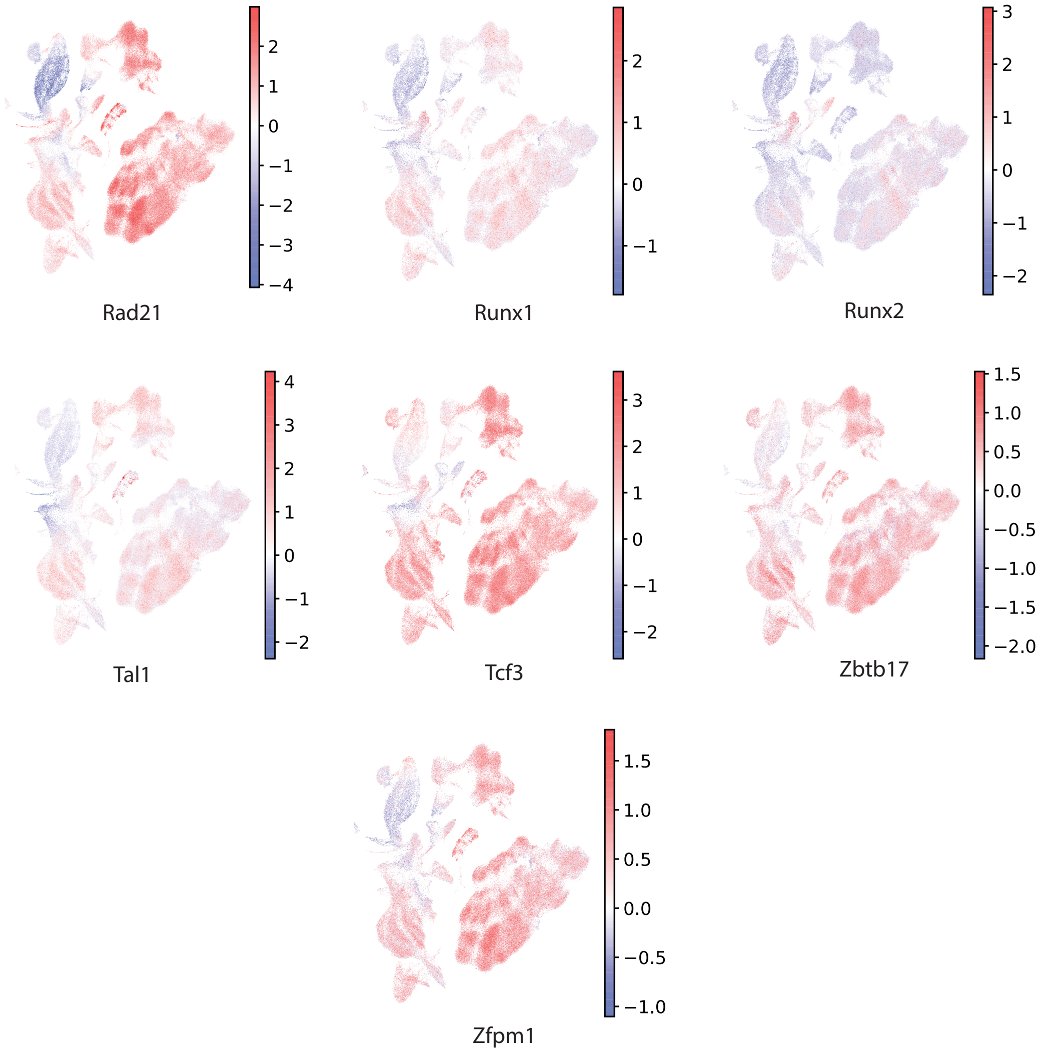

Appendix Figures S8-13.

DoT scores calculated using genes differentially expressed after loss of indicated TFs in Hoxb8-FL cells in the context of the mouse LK/LSK or human BMMC landscapes.
