# Supplementary material for: Interactions between lineage‐associated transcription factors govern haematopoietic progenitor states
Source: EMBO J. 2020 Oct 26;39(24):e104983. doi: 10.15252/embj.2020104983 (PMC7737608; doi:10.15252/embj.2020104983)
Supplement: Supplementary file 2 — Expanded View Figures PDF [file EMBJ-39-e104983-s002.pdf]

Expanded View Figures

Figure EV1. Parameters critical for the CRISPR/Cas9 screen.

A Flow cytometry analysis of Hoxb8-FL cells transduced with sgRNA targeting CD45 (the Ptprc gene). Successfully transduced cells are BFP^+, and mutant cells are BFP^+, $CD45^-$. Ptprc is successfully mutated in 48% of transduced cells, whereas almost all non-transduced cells remain $CD45^+$.

B High-throughput sequencing analysis of genomic DNA reads with frameshift mutations at predicted cutting sites following treatment of Hoxb8-FL cells with 11 different sgRNAs.

C Experimental design applied to screening of 38 transcription factors, each gene was targeted with 3 sgRNAs in 8 replicates. Two sets of controls were used: sgRNA targeting the Rosa26 locus and sgRNA targeting a GFP sequence (absent in the genome). Hoxb8 ectopic expression was disabled by β-oestradiol withdrawal.

D R^2 values for observed changes in expression for each pair of sgRNAs targeting the same gene (using genes differentially expressed in 2 out of 3 comparisons).

E A heatmap representing genes differentially expressed between the Gata3 sgRNA treated and control cells at all assayed time-points. The signature observed in the first three time-points disappears from 7 days onwards. Fraction of intronic reads is displayed above the heatmap. Barplot below shows the number of differentially expressed genes at each time-point.

F Related to (D), an example of correlation in gene expression changes across three sgRNAs targeting Gata3 sgRNA. Analysis performed using genes differentially expressed in at least 2 out of 3 comparisons. Blue line indicates the linear fit with shaded areas as confidence intervals.

G Relative survival analysis of cells transduced with sgRNAs against Cebpa, Gata3 and Myc. Control cells treated with sgRNAs targeting GFP or Rosa26 loci indicate background fluorescent population changes, with only a small loss of the positive population. The fraction of BFP^+ has been normalised to a parallel control performed in Hoxb8 cells not expressing the Cas9 protein. Error bars—standard error of the mean. R26—4 replicates, GFP—2 replicates, Cebpa, Gata3, Myc—3 replicates per condition.

© 2020 The Authors Published under the terms of the CC BY 4.0 license

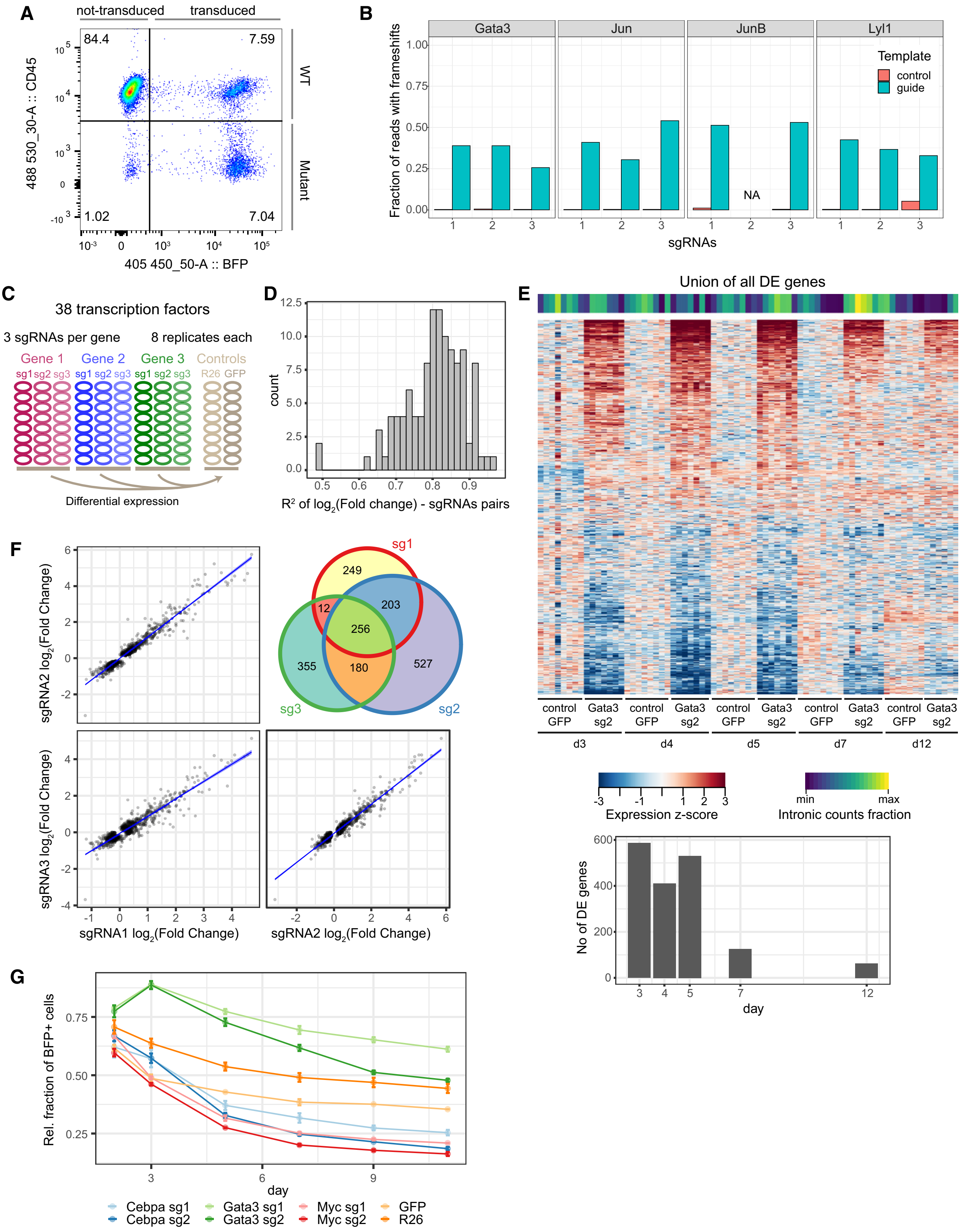
A
not-transduced
transduced
84.4
7.59
1.02
7.04
WT
Mutant
488 530_30-A :: CD45
405 450_50-A :: BFP
B
Gata3
Jun
JunB
Lyl1
Template
control
guide
NA
Fraction of reads with frameshifts
sgRNAs
C
38 transcription factors
3 sgRNAs per gene
8 replicates each
Gene 1
Gene 2
Gene 3
Controls
R26 GFP
Differential expression
D
count
R² of log2(Fold change) - sgRNAs pairs
E
Union of all DE genes
control GFP
Gata3 sg2
d3
d4
d5
d7
d12
Expression z-score
min
max
Intronic counts fraction
No of DE genes
day
F
sgRNA2 log2(Fold Change)
sgRNA3 log2(Fold Change)
sgRNA1 log2(Fold Change)
sg1
sg2
sg3
249
203
12
256
355
180
527
G
Rel. fraction of BFP+ cells
day
Cebpa sg1
Cebpa sg2
Gata3 sg1
Gata3 sg2
Myc sg1
Myc sg2
GFP
R26

Figure EV1.

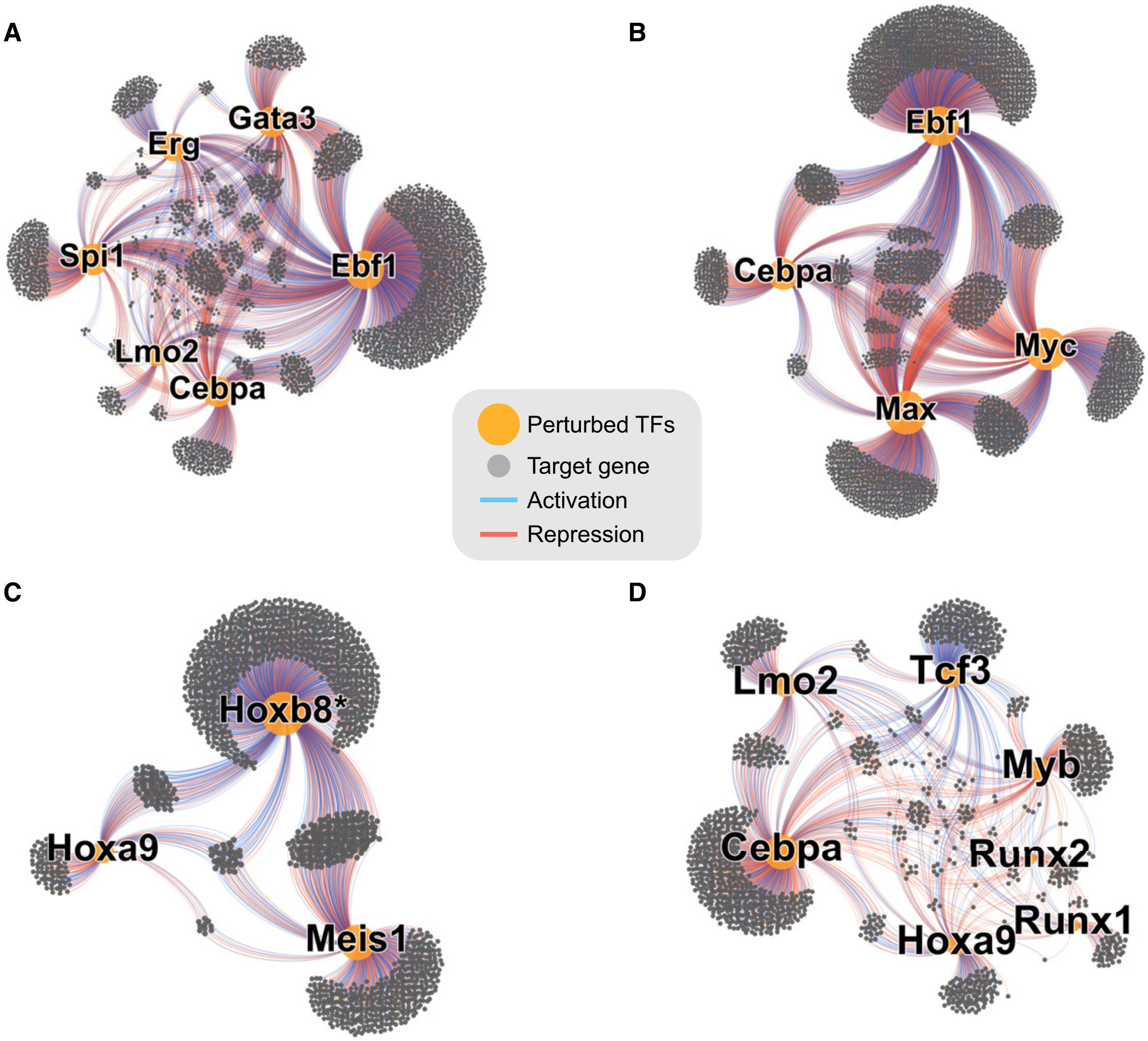

Figure EV2. Key TF subnetworks controlling the progenitor state.

A–D Subgraphs isolated from Fig 2B. In each panel, the indicated TFs were isolated together with their downstream targets.

Figure EV3. Detailed view of the interactions between Cebpa/Meis1/Spi1 and Hoxb8.

A, B Number of genes per binary combination of expression change directions for TF1 perturbation (Cebpa, Meis1 or Spi1), Hoxb8* perturbation and their interaction. Classified as shown in Fig 4C. Low stringency (A)—only genes coregulated by separate Cebpa/Meis1/Spi1 and Hoxb8 perturbations (FDR < 0.1 and $|\log_2(\text{fold change})| > 0.2$). Direction of expression changes was classified as $|\log_2(\text{fold change})| > 0.2$. High stringency (B)—only genes with a significant interaction term (FDR < 0.1 and $|\log_2(\text{fold change})| > 0.2$) and expression change directions classified as FDR < 0.1 and $|\log_2(\text{fold change})| > 0.2$.

C Venn diagrams depicting number of genes with significant terms, as categorised in (B).

 © 2020 The Authors Published under the terms of the CC BY 4.0 license

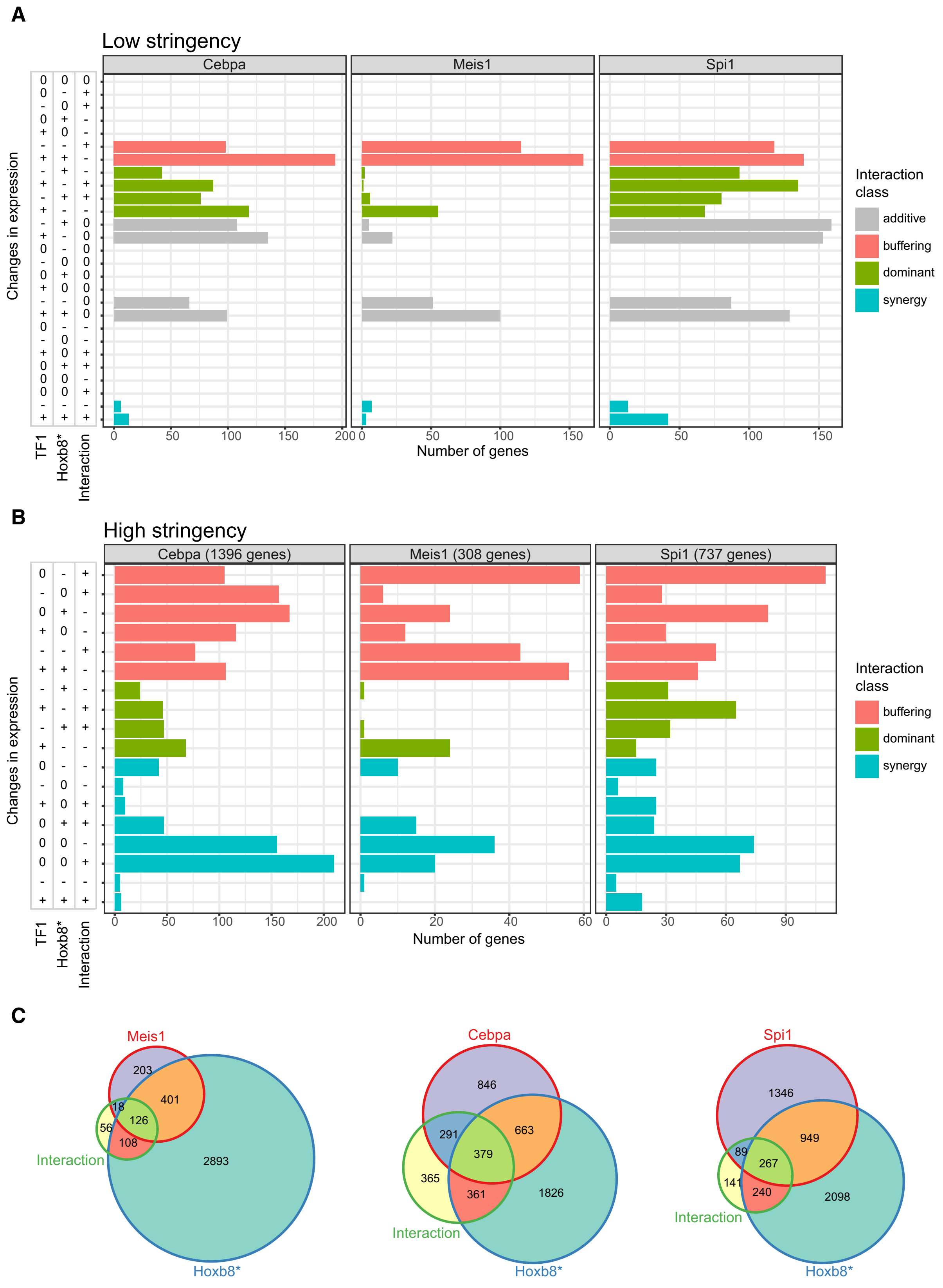

Figure EV3.

© 2020 The Authors Published under the terms of the CC BY 4.0 license

Figure EV4. Ebf1 function and regulatory programme maintaining a self-renewing, multipotent state.

- A Relative survival analysis of cells transduced with sgRNAs against Ebf1 or Myc. Control cells treated with sgRNAs targeting GFP or Rosa26 loci indicate background fluorescent population changes, with only a small loss of the positive population. The fraction of BFP^+ has been normalised to a parallel control performed in Hoxb8 cells not expressing the Cas9 protein. Error bars indicate standard error of the mean. R26—4 replicates, GFP—2 replicates, Myc and Ebf1—3 replicates per condition.
- B Cell size approximated by the FSC parameter for data in (A), error bars indicate standard error of the mean. R26—4 replicates, GFP—2 replicates, Myc and Ebf1 3 replicates per condition.
- C A heatmap showing relative similarity of Ebf1 binding sites across a range of other cell types, including B-cell progenitor states. Please note a substantial similarity to the early EBf1 peaks identified in (Li *et al*, 2018).
- D Overlap of genes differentially expressed following Ebf1 loss in Hoxb8-FL cells and genes changing expression within 24 h of re-expression of Ebf1 in $Ebf1^{-/-}$ pre-pro-B cells (Li *et al*, 2018).
- E Overlap of sets in (D) and top 500 genes contributing to the myeloid programme downstream of Ebf1 (DoT score after Ebf1 loss, see Fig EV5F) within the neutrophil progenitor cluster in the LK/LSK landscape (dark blue colour in Fig 7B).
- F Correlation in expression changes following Ebf1 loss in Hoxb8-FL cells and re-expression of Ebf1 (24 h) in $Ebf1^{-/-}$ pre-pro-B cells but subset for 138 Myo genes from (E). Blue line indicates the linear fit with shaded areas as confidence intervals.
- G Comparison of Ebf1, Gata3, Pax5 and Cebpa expression levels among: Hoxb8-FL cells, pre-pro-B cells resuming differentiation (purple) (Li *et al*, 2018) and early lymphoid/B-cell progenitors from (Revilla-i-Domingo *et al*, 2012) including Pax5-deficient pro-B cells. *Please note that Ebf1 and Pax5 RNA levels do not reflect functional gene products in the respective knockout setting. Horizontal line indicates average expression.
- H Expression of the key marker genes along pseudotime corresponding to early lymphoid/B-cell differentiation trajectory in mouse bone marrow. Data from Loughran *et al* (2017). Colour bar indicates immunophenotypic identity for each cell analysed (ordered along pseudotime). LMPP—lymphoid-primed multipotent progenitors, ALP—all-lymphoid progenitor, BLP—B cell-biased common lymphoid precursor.
- I Expression of Ebf1, Pax5, Gata3 and Cebpa along pseudotime corresponding to early lymphoid/B-cell differentiation trajectory in mouse bone marrow. Data from Loughran *et al* (2017).

Data information: References in Fig EV4: 1—(Li *et al*, 2018), 2—(Treiber *et al*, 2010), 3—(Ungerbäck *et al*, 2015), 4—(Hu *et al*, 2016), 5—(van Oevelen *et al*, 2015), 6—(Collombet *et al*, 2017), 7—(Boller *et al*, 2016), 8—(Györy *et al*, 2012), 9—(Griffin *et al*, 2013), 10—(Vilagos *et al*, 2012).

 © 2020 The Authors Published under the terms of the CC BY 4.0 license

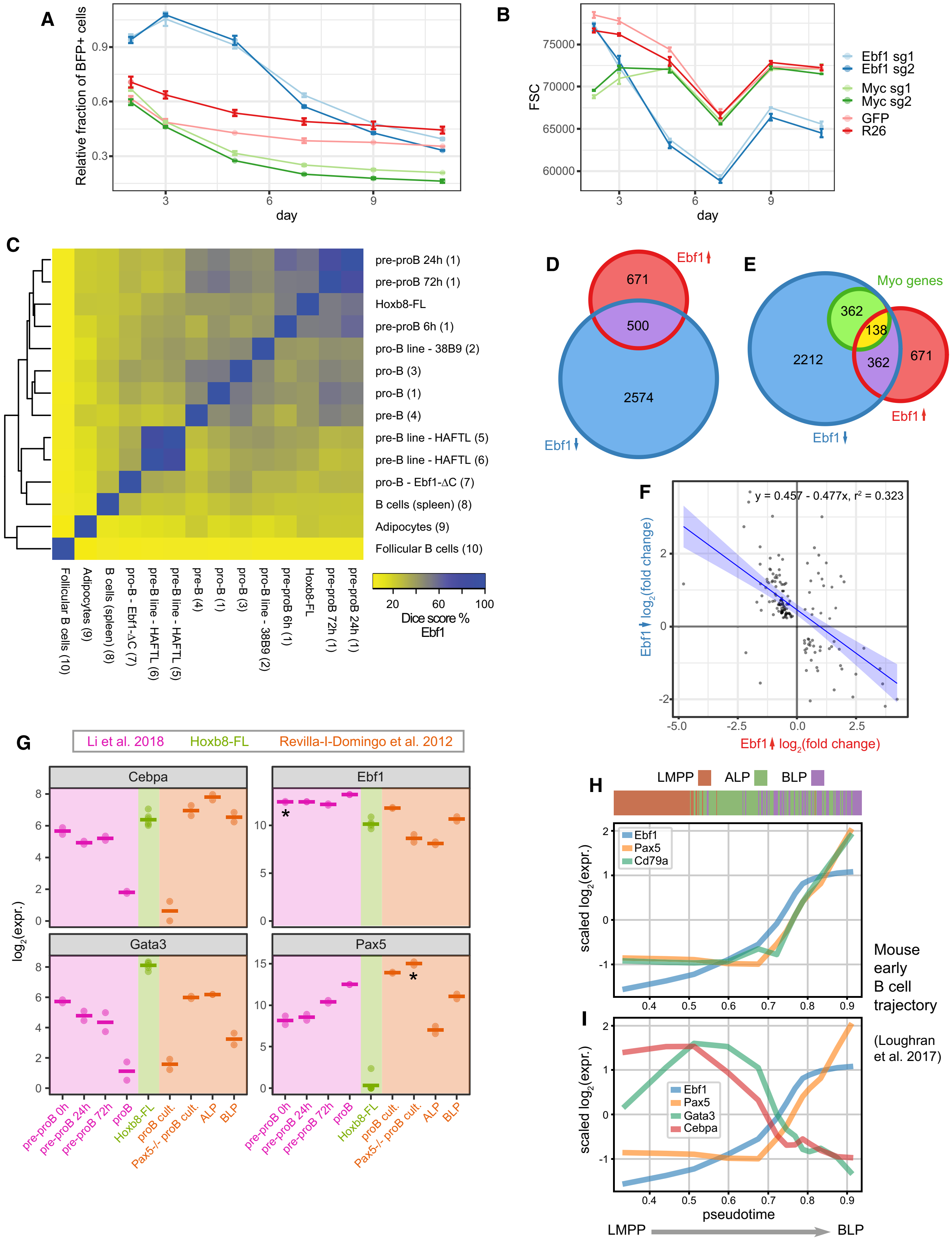
A
Relative fraction of BFP+ cells
day
B
FSC
day
Ebf1 sg1
Ebf1 sg2
Myc sg1
Myc sg2
GFP
R26
C
pre-proB 24h (1)
pre-proB 72h (1)
Hoxb8-FL
pre-proB 6h (1)
pro-B line - 38B9 (2)
pro-B (3)
pro-B (1)
pre-B (4)
pre-B line - HAFTL (5)
pre-B line - HAFTL (6)
pro-B - Ebf1-ΔC (7)
B cells (spleen) (8)
Adipocytes (9)
Follicular B cells (10)
Dice score %
Ebf1
D
Ebf1
671
500
2574
E
Myo genes
362
138
2212
362
671
F
y = 0.457 - 0.477x, r² = 0.323
Ebf1 log2(fold change)
G
Li et al. 2018
Hoxb8-FL
Revilla-I-Domingo et al. 2012
Cebpa
Ebf1
Gata3
Pax5
log2(expr.)
pre-proB 0h
pre-proB 24h
pre-proB 72h
proB
proB cult.
Pax5-/- proB cult.
ALP
BLP
H
LMPP
scaled log2(expr.)
Cd79a
Mouse early B cell trajectory
(Loughran et al. 2017)
I
pseudotime

Figure EV4.

Figure EV5. DoT score analysis helps interpreting TF functions.

A–F DoT scores calculated using genes differentially expressed after loss of Cebpa, Spi1, Gata3, Ebf1, Hoxa9 and Meis1 in Hoxb8-FL cells in the context of the mouse LK/LSK (top) or human BMMC (bottom) landscapes.

 © 2020 The Authors Published under the terms of the CC BY 4.0 license

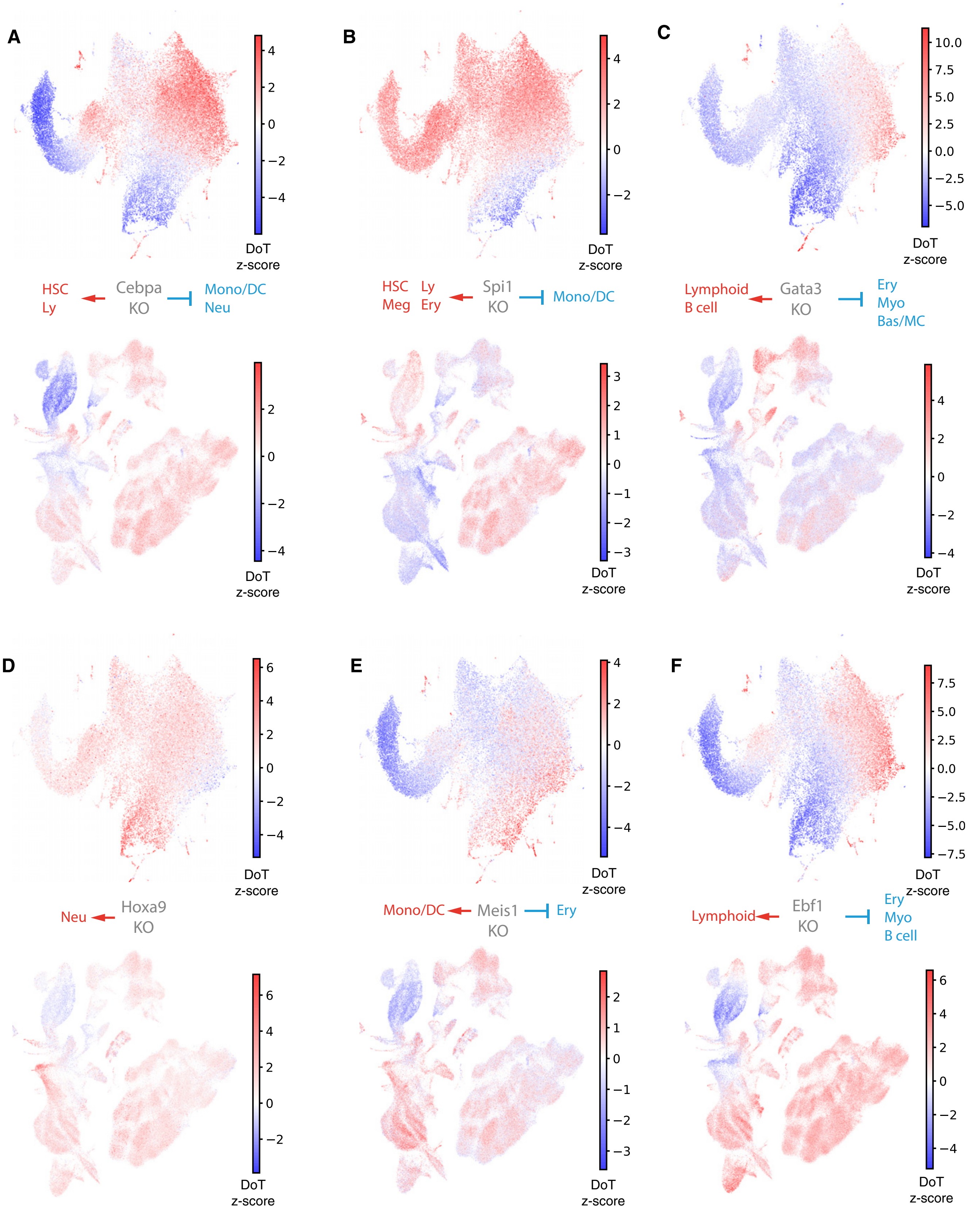
A
DoT
z-score
HSC
Ly
Cebpa
KO
Mono/DC
Neu
B
HSC Ly
Meg Ery
Spi1
KO
Mono/DC
C
Lymphoid
B cell
Gata3
KO
Ery
Myo
Bas/MC
D
Neu
Hoxa9
KO
E
Mono/DC
Meis1
KO
Ery
F
Lymphoid
Ebf1
KO
Ery
Myo
B cell

Figure EV5.
